# Endothelin Type A Receptor Genotype is a Determinant of Quantitative Traits of Metabolic Syndrome in Asian Hypertensive Families: A SAPPHIRe Study

**DOI:** 10.3389/fendo.2013.00172

**Published:** 2013-11-28

**Authors:** Low-Tone Ho, Yung-Pei Hsu, Chin-Fu Hsiao, Chih-Tai Ting, Kuang-Chung Shih, Lee-Ming Chuang, Kamal Masaki, John Grove, Thomas Quertermous, Chi-Chung Juan, Ming-Wei Lin, Shu-Chiung Chiang, Yii-Der I. Chen

**Affiliations:** ^1^Department of Medical Research and Education, Taipei Veterans General Hospital, Taipei, Taiwan; ^2^School of Medicine, National Yang-Ming University, Taipei, Taiwan; ^3^Faculty of Medicine, National Yang-Ming University, Taipei, Taiwan; ^4^Institute of Physiology, National Yang-Ming University, Taipei, Taiwan; ^5^Institute of Clinical Medicine, National Yang-Ming University, Taipei, Taiwan; ^6^Division of Biostatistics and Bioinformatics, National Health Research Institutes, Taipei, Taiwan; ^7^Cardiovascular Center, Taichung Veterans General Hospital, Taichung, Taiwan; ^8^Division of Endocrinology and Metabolism, Tri-Service General Hospital, Taipei, Taiwan; ^9^Department of Internal Medicine, National Taiwan University Hospital, Taipei, Taiwan; ^10^Graduate Institute of Clinical Medicine, National Taiwan University, Taipei, Taiwan; ^11^Kuakini Medical Center, Honolulu, Hawaii; ^12^Department of Public Health Sciences and Epidemiology, John A. Burns School of Medicine, University of Hawaii and Pacific Health Research Institute, Honolulu, Hawaii; ^13^Division of Cardiovascular Medicine, Falk Cardiovascular Research Center, Stanford University, Stanford, CA, USA; ^14^Information Service Center, Taipei Veterans General Hospital, Taipei, Taiwan; ^15^Medical Genetics Institute, Cedars-Sinai Medical Center, University of California at Los Angeles, Los Angeles, CA, USA

**Keywords:** ET_A_ receptor, T323C polymorphism, metabolic syndrome, hypertension

## Abstract

Co-heritability of hypertension and insulin resistance (IR) within families not only implies genetic susceptibility may be responsible for these complex traits but also suggests a rational that biological candidate genes for hypertension may serve as markers for features of the metabolic syndrome (MetS). Thus we determined whether the T323C polymorphism (rs5333) of endothelin type A (ET_A_) receptor, a predominant receptor evoking potent vasoconstrictive action of endothelin-1, contributes to susceptibility to IR-associated hypertension in 1694 subjects of Chinese and Japanese origins. Blood pressures (BPs) and biochemistries were measured. Fasting insulin level, insulin-resistance homeostasis model assessment (HOMA_IR_) score, and area under curve of insulin concentration (AUC_INS_) were selected for assessing insulin sensitivity. Genotypes were obtained by methods of polymerase chain reaction-restriction fragment length polymorphism. Foremost findings were that minor allele frequency of the T323C polymorphism was noticeable lower in our overall Asian subjects compared to multi-national population reported in gene database; moreover both the genotypic and allelic frequencies of the polymorphism were significantly different between the two ethnic groups we studied. The genotype distributions at TT/TC/CC were 65, 31, 4% in Chinese and 51, 41, 8% in Japanese, respectively (*p* < 0.0001). Additionally, carriers of the C homozygote revealed characteristics of IR, namely significantly higher levels of fasting insulin, HOMA_IR_ score, and AUC_INS_ at 29.3, 35.3, and 39.3%, respectively, when compared to their counterparts with TT/TC genotypes in Chinese. Meanwhile, the CC genotype was associated with a higher level of high density lipoprotein cholesterol in Japanese. No association of the polymorphism with BP was observed. This study demonstrated for the first time that T323C polymorphism of ET_A_ receptor gene was associated with an adverse insulin response in Chinese and a favorite atherogenic index in Japanese.

## Introduction

Insulin resistance (IR) and hypertension are related traits. Individuals who are insulin resistant are glucose intolerant, hyperinsulinemic, have higher blood pressure (BP) as well as atherogenic dyslipidemia (1). The clustering of these cardiovascular disease (CVD) risks associated with IR displayed familial characteristics as well, often observed in diabetes and hypertension families (2–5). For example, path analysis showed co-heritability of BP and IR in Hispanic subjects with hypertension parents (6). Cosegregation of traits in family not only reveals genetic susceptibility but also implies genes contribute to one will predict other traits in the family members. Therefore, biological candidate genes for hypertension may serve as genetic markers for features of the metabolic syndrome (MetS) (7). Dysfunction of endothelin system (ETS) is one of ancillary clinical features of the MetS that could lead to abnormalities in cardiovascular structure and function.

Endothelins are a family of three peptides of 21 amino acids with strong vasoconstrictor effects (8, 9). Two receptors have been cloned, endothelin type A (ET_A_) and endothelin type B receptor (ET_B_) which bind the three endothelins with various affinities (10). Endothelin-1 (ET-1) and nitric oxide are the prototypes of endothelium-derived contracting factor and relaxing factor, respectively, and their effects on the cardiovascular system have been studied in depth (11–14). Experimental studies have provided evidence that ET-1 may exert a bidirectional vascular tonicity effect either by vasodilation through enhancing nitric oxide production via ET_B_ receptors located in endothelial cells, or by vasoconstriction via activating of ET_A_ receptors prevalently located in the vascular smooth muscle cells and/or blunting ET_B_ receptors. Moreover, findings from both clinical and animal studies are in favor of a direct role of ET-1 in the pathogenesis of hypertension (15). In addition, we and others have previously reported in animal and cellular experiments showing that ET-1 and ET_A_ receptor probably played a significant role in the pathogenesis of IR and its association with hypertension (16–19). However, the link between ETS and IR-associated hypertension in clinical settings remain to be elucidated.

The diverse expression pattern of the ETS components is associated with a complex pharmacology and its counteracting physiological actions. New modulators of the ETS have been described: retinoic acid (20), leptin (21), prostaglandins and hypoxia (22). Endothelins can be considered as regulators working in paracrine and autocrine fashion in a variety of organs in different cellular types. They affect the physiology and pathophysiology of the liver, muscle, skin, adipose tissue, and reproductive tract. In contrast, the genetic determinants of ETS and their association to the metabolic CVD risks have not been extensively studied. Previous findings (23–30) on associations of some genetic polymorphisms of endothelins and their receptors with BP were controversy; moreover, few features of MetS were ascertained in these studies (30).

The present study was investigated to address the genetic polymorphism of ET_A_ receptor in a large Chinese and Japanese cohort with hypertensive proband. We hypothesized that ET_A_ receptor, a component of the ETS shown to play essential roles in the maintenance of renal homeostasis and basal vascular tone, may confer susceptibility to IR-related hypertension due to its multiple expression sites and complex modulators. We demonstrated that ET_A_ receptor exon 6 T323C polymorphism (rs5333) was associated with IR and hyperinsulinemia in Chinese, and was related with high density lipoprotein (HDL)-cholesterol and atherogenic index in Japanese.

## Materials and Methods

### Study subjects

Subjects in this current study were those for the Stanford Asian-Pacific Program in Hypertension and Insulin Resistance (SAPPHIRe) genetic study that aimed at mapping genetic loci for hypertension and IR in a well-characterized population regarding IR and its related metabolic traits. The study design and subject recruitment for SAPPHIRe were described in detail previously (31). In brief, SAPPHIRe, a family based and sibling-paired design, incorporated both concordant sibling pairs (i.e., two or more hypertensive full siblings) and discordant sib-pairs (i.e., hypertensive and hypo-normotensive siblings). Probands were hypertensive individuals with Chinese or Japanese descent. The BP values at the 80th percentile or more were considered as hypertension, which was equivalent to systolic BP of at least 160 or diastolic BP of at least 95 mmHg or taking two or more medications for high BP. The hypo-normotensive sib controls was a combination of subjects whose BP values below the bottom 30th percentile (i.e., hypotensive) and whose BP not qualified for hyper- or hypotensive-status (i.e., normotensive). In this particular study, we included in the analysis only the non-diabetic subjects (fasting plasma glucose <126 mg/dl) to reduce the confounding effect of hyperglycemia on the quantitative measurements of glucose homeostasis. Consequently, the study population genotyped comprised of 929 hypertension and 765 hypo-normotension. Written informed consent from all participants was obtained and the study protocol was approved by the Institutional Review Board of each field center.

### Phenotypic and biochemical characterization

SAPPHIRe study incorporated physical examinations and anthropometric measurements, routine clinical laboratory tests, an oral glucose tolerance test (OGTT), and administration of an informative questionnaire to obtain biochemical data and phenotypic information on subjects. Measurement protocols for BP and anthropometry were previously reported in detail (5, 32). Body mass index (BMI) was calculated as body weight in kilograms divided by body height square in meters. A standard dose of 75-g glucose monohydrate dissolved in water was administered to the study subjects after an overnight fast. Venous blood was sampled before and 1- and 2-h after oral glucose challenge, and plasma was prepared by immediate centrifugation. Plasma glucose was measured by a glucose oxidase method and plasma insulin was determined using an enzymatic immunoassay. Concentrations of fasting plasma glucose and insulin obtained from OGTT were applied to calculate insulin-resistance homeostasis model assessment (HOMA_IR_), a surrogate index for insulin sensitivity validated to the reference method of the euglycemic hyperinsulinemic clamp (33), using the formula as described by Matthews et al. (34): fasting insulin (μU/mL) × fasting plasma glucose (mmol/L)/22.5. High HOMA_IR_ scores denoted low insulin sensitivity, i.e., more insulin resistant. Three surrogate indices, fasting insulin level, HOMA_IR_ score, area under curve of insulin concentration (AUC_INS_), were selected for assessing insulin sensitivity. Clinical chemistries included fasting concentrations on total cholesterol, triglycerides, HDL cholesterol were determined by automated enzymatic methods using colorimetry as end point. Low density lipoprotein (LDL)- and very low density lipoprotein (VLDL)-cholesterol were calculated by Friedewald equation (35). All lipid determinations were standardized with the centers for disease control and prevention/national heart, lung, and blood institute (CDC/NHLBI) lipid standardization program and further assured by participating in the College of American pathologists (CAP) survey.

### Genotyping of ET_A_ receptor gene polymorphism

Using polymerase chain reaction (PCR) followed by restriction fragment length polymorphism (RFLP) analysis, we determined the polymorphism of exon 6 codon 323 T → C in the ET_A_ receptor gene. The DNA fragment containing the polymorphism site was amplified using the forward primer 5′-TTTCTCACTTTCCTTTAGCG-3′ and the reverse primer 5′-ACCTAAGTAATTCACATCGG-3′. PCR amplifications were carried out in a final volume of 30 μL by 30 cycles at 94, 50, and 72°C each for 30 s and followed by a final extension step at 72°C for 10 min. Blank controls containing no genomic DNA were included in each run of PCR amplification to monitor contamination. Agarose gel electrophoresis on randomly selected PCR products was performed routinely to check if amplification succeeded. For subsequent RFLP analysis, the PCR product was mixed with 1U *Afl*II and 1 μg/mL BSA in the appropriate buffer (New England Biolabs, Beverly, MA, USA) and incubated at 37°C for 3 h to ensure the complete enzyme digestion. The digested products were separated by electrophoresis on 3% agarose gel, visualized by ethidium bromide staining and analyzed under UV light. The person who assessed the genotype was blinded to the clinical data of the subjects from whom the samples originated. Direct DNA sequencing using ABI PRISM377 machine together with BigDye™ Terminator Cycle Sequencing Ready Reaction Kit (Perkin Elmer, Applied Biosystems, Foster City, CA, USA) on randomly selected PCR products from each genotype was also performed to validate the polymorphisms.

The restriction enzyme *Afl*II is specific for the sequence C/TTAAG. Digestion of PCR product with *Afl*II resulted in two fragments of different sizes at 67 and 87 bp for the existence of C allele. However, the restriction site is lost in the presence of T variant, and therefore digestion products shown a single band at 154 bp. Thus, three genotypes TT, TC, and CC at codon 323 in exon 6 of ET_A_ receptor gene were defined according to numbers of fragments by RFLP, depending on the presence of C allele as a restriction site for *Afl*II.

### Statistical analyses

Data were expressed as the mean and the standard deviation (mean ± SD), unless otherwise specified. The ET_A_ receptor genotype distributions and allele frequencies between subjects with different ethnicity and BP status were compared by Chi-square analyses. The Hardy–Weinberg equilibrium was also examined using the Chi-square test. The association of the ET_A_ receptor gene variant with the variables of interest was assessed using mixed model regression analyses as described previously (36), with familial effect controlled by adjusting for family clustering effect. The mean differences of metabolic variables between siblings discordant for the genotypes within families were further evaluated by the delta method in which the potential family random effect was eliminated (5, 36). Specifically, due to genetic dependence of siblings, differences in the phenotypic traits with regard to genotypes were compared by paired *t* tests, while pooling the T allele carriers together, before and after adjusting for possible confounding variables including age, gender, and BMI. All analyses were performed using the SAS statistical software package (SAS Institute Inc., Cary, NC, USA), and tests were two-sided throughout and a *p*-value of <0.05 was considered statistically significant.

## Results

The general characteristics of the participants categorized by ethnicity are shown in Table 1. No significant differences were found in gender distribution and age onset for hypertension between the two ethnic groups. Chinese subjects were somewhat younger in age, had shorter duration in hypertension, and exhibited a smaller waist girth and lower BMI, and waist/hip ratio (WHR) than the Japanese. Japanese possessed elevated levels of blood lipids except for LDL-cholesterol and resulted in a decreased ratio of total- to HDL-cholesterol as compared to the Chinese. There were no consistent differences in the glucose homeostasis parameters such as fasting and postprandial glucose and insulin, and the surrogate insulin indices.

**Table 1:** **The anthropometric and metabolic characteristics of study population (mean ± SD)**.

Variable	Chinese *N* = 1304	Japanese *N* = 390	All subjects *N* = 1694	*p*-Value
Age (year)	49.1 ± 8.6	55.3 ± 8.3	50.5 ± 8.9	<0.0001
Gender (male/female, %)	44.8/55.2	41.0/59.0	43.9/56.1	NS
HTN [*n*, (%)]	663 (50.8)	266 (68.2)	929 (54.8)	<0.0001
Age of HTN onset (year)	43.9 ± 9.5	43.0 ± 9.8	43.6 ± 9.6	NS
Duration of HTN (year)	7.2 ± 8.1	12.9 ± 10.2	8.8 ± 9.1	<0.0001
Waist (cm)	84.2 ± 10.3	89.4 ± 11.4	85.4 ± 10.8	<0.0001
BMI (kg/m^2^)	25.2 ± 3.4	26.4 ± 3.9	25.5 ± 3.6	<0.0001
WHR (cm/cm)	0.87 ± 0.08	0.92 ± 0.09	0.88 ± 0.08	<0.0001
Blood pressure (mmHg)
Systolic	128.8 ± 24.5	131.9 ± 20.4	129.5 ± 23.7	0.0316
Diastolic	76.9 ± 13.5	77.6 ± 11.6	77.1 ± 13.2	NS
Lipid profiles (mg/dl)
Triglycerides	124.2 ± 76.3	162.4 ± 99.9	132.3 ± 83.3	<0.0001
Total CHOL	187.3 ± 38.6	200.0 ± 36.3	190.0 ± 38.5	<0.0001
HDL-cholesterol	44.3 ± 12.2	49.9 ± 14.8	45.5 ± 13.0	<0.0001
VLDL-cholesterol	24.2 ± 13.3	30.0 ± 15.0	25.4 ± 13.9	<0.0001
LDL-cholesterol	118.8 ± 36.3	118.7 ± 32.0	118.8 ± 35.4	NS
Atherogenic index (CHOL/HDL)	4.5 ± 1.5	4.3 ± 1.3	4.5 ± 1.4	0.0106
Oral glucose tolerance test
Plasma glucose (mg/dl)
Fasting	90.2 ± 11.3	96.1 ± 10.7	91.4 ± 11.4	<0.0001
1-h	173.8 ± 44.1	165.6 ± 47.4	172.4 ± 44.8	0.0090
2-h	138.8 ± 44.2	140.3 ± 43.6	139.1 ± 44.1	NS
Plasma insulin (μIU/ml)
Fasting	7.6 ± 5.5	7.2 ± 5.0	7.6 ± 5.4	NS
1-h	77.5 ± 59.3	64.3 ± 44.2	75.3 ± 57.2	0.0010
2-h	66.7 ± 60.1	65.5 ± 51.2	66.5 ± 58.5	NS
Insulin resistance indices
HOMA_IR_	1.8 ± 1.4	1.8 ± 1.3	1.8 ± 1.4	NS
AUC_INS_ (μIU/ml/h)	114.8 ± 82.7	100.3 ± 63.9	112.4 ± 80.0	0.0100

*HTN, hypertension; BMI, body mass index; WHR, waist-to-hip ratio; CHOL, cholesterol; HDL, high density lipoprotein; VLDL, very low density lipoprotein; LDL, low density lipoprotein; HOMA_IR_, insulin-resistance homeostasis model assessment; AUC_INS_, area under curve of insulin concentration*.

A total of 1694 biethnic sibling subjects (1304 Chinese and 390 Japanese) deriving from eligible families were analyzed. The genotype distributions in overall or each for Chinese and Japanese were all in compliance with the Hardy–Weinberg equilibrium (*p* = 0.166 for overall, *p* = 0.225 for Chinese, and *p* = 0.949 for Japanese). Significant differences (*p* < 0.0001) on genotype frequencies were observed between subjects with different ethnic backgrounds (Table 2). The C homozygote was present only in 4.4% of the Chinese but nearly doubled in 8.5% of the Japanese, whereas the more common T homozygote was at 64.9 and 50.5% for Chinese and Japanese, respectively. The relative frequencies of the T and C alleles of the ET_A_ receptor gene in Chinese were 0.80 and 0.20, and in Japanese 0.71 and 0.29 (*p* < 0.0001). Neither genotype nor allele frequency was different with respect to gender. Moreover, there was no statistical significant difference in genotypes concerning the BP status in our studied subjects despite the anticipated role of ET_A_ receptor in the regulation of cardiovascular complications.

**Table 2:** **Distribution of genotypes and alleles for the ETAR T323C polymorphism in studied subjects**.

Subjects	Genotype, *n* (%)			Allele %	
	TT	TC	CC	T	C
Ethnicity*
Chinese	846 (64.9)	400 (30.7)	58 (4.4)	80.2	19.8
Japanese	197 (50.5)	160 (41.0)	33 (8.5)	71.0	29.0
BP status
Hypertension	556 (59.8)	323 (34.8)	50 (5.4)	77.2	22.8
Hypo-normotension	487 (63.6)	237 (31.0)	41 (5.4)	79.2	20.8
Overall subjects of eligible families	1043 (61.6)	560 (33.0)	91 (5.4)	78.1	21.9

*p < 0.0001.

**Figure 1:**
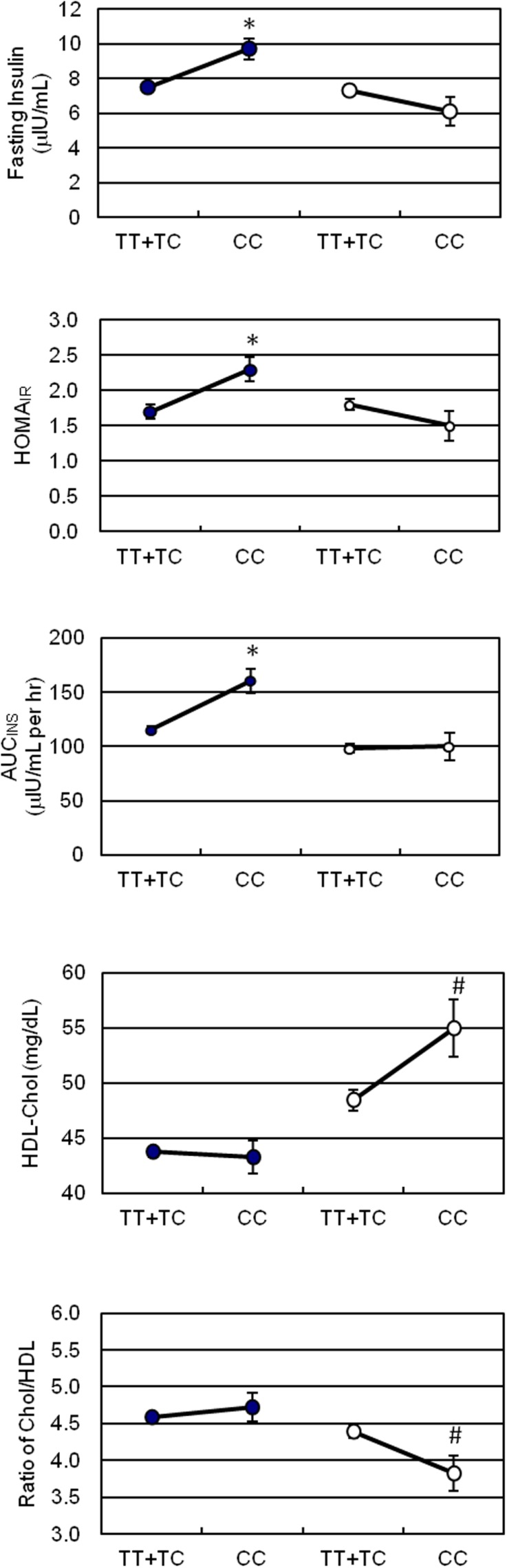
**Effect of the ET_A_ receptor genotypes on surrogate indices of insulin sensitivity, HDL-cholesterol, and atherogenic index**. -∙- for Chinese, -∘- for Japanese. Vertical lines represent SD. Significant differences denote as *p < 0.01, ^#^*p* < 0.05.

Due to the significant ethnic differences, we next evaluated the association of genotypes of ET_A_ receptor with the quantitative traits of the MetS in each individual ethnicity. Using a mixed model controlling for family effect, we observed association of ET_A_ receptor genotypes with fasting and postprandial insulin concentrations, as well as HOMA_IR_ and AUC_INS_ indices in Chinese population before and after adjusting for confounding variables (Table 3). In the Japanese cohort, ET_A_ receptor genotype was significantly associated with the VLDL- and HDL-cholesterol concentrations and consequently with the ratio of total to HDL cholesterol (Table 4). The adjustment for age, gender, and BMI did not affect the observations but strengthened the association seen in both Chinese and Japanese. To clearly illustrate the discrete nature of the association of ET_A_ receptor genotype with MetS in Chinese and Japanese, comparisons on the mean values of the quantitative traits with significant differences were made and presented in Figure 1. Because the initial cohort was ascertained as a family study, the genetic association of ET_A_ receptor alleles with variables of the MetS was further analyzed by delta method. The results were essentially unchanged and these analyses further confirmed the association of ET_A_ receptor gene with the CVD risks of IR in Chinese (Data not shown).

**Table 3:** **Clinical characteristics of the Chinese siblings with discordant ET_A_ receptor genotypes are compared with or without adjustment for age, gender, and BMI (Mean ± SD)**.

Traits	Genotypes		Unadjusted *p*-value	Adjusted *p*-value
	TT/TC	CC	
Subject number	1215	57		
Blood pressure (mmHg)
Systolic	129.4 ± 0.9	129.5 ± 3.5	0.8799	0.9693
Diastolic	77.3 ± 0.5	77.9 ± 1.9	0.8995	0.7861
Lipid profiles (mg/dl)
Triglycerides	126.1 ± 2.7	123.8 ± 10.7	0.4184	0.8297
Total CHOL	187.8 ± 1.5	188.1 ± 5.3	0.6661	0.9580
HDL-cholesterol	43.7 ± 0.4	43.3 ± 1.5	0.0600	0.7614
VLDL-cholesterol	24.5 ± 0.5	24.8 ± 1.8	0.2227	0.9023
LDL-cholesterol	119.6 ± 1.4	117.3 ± 5.0	0.4414	0.6473
Atherogenic index (CHOL/HDL)	4.6 ± 0.1	4.7 ± 0.2	0.0951	0.4653
Oral glucose tolerance test
Plasma glucose (mg/dl)
Fasting	90.4 ± 0.4	92.4 ± 1.5	0.1395	0.1690
1-h	173.7 ± 1.7	181.9 ± 6.0	0.1148	0.1755
2-h	138.8 ± 1.7	139.2 ± 6.0	0.8998	0.9482
Plasma insulin (μIU/ml)
Fasting	7.5 ± 0.2	9.7 ± 0.6	0.0055	0.0007
1-h	76.9 ± 2.1	107.7 ± 8.3	0.0004	0.0003
2-h	68.1 ± 2.4	94.3 ± 8.3	0.0054	0.0017
Insulin resistance indices
HOMA_IR_	1.7 ± 0.1	2.3 ± 0.2	0.0070	0.0014
AUC_INS_ (μIU/ml/h)	115.1 ± 3.1	160.3 ± 11.3	<0.0001	<0.0001

*CHOL, cholesterol; HDL, high density lipoprotein; LDL, low density lipoprotein; VLDL, very low density lipoprotein, HOMA_IR_, insulin-resistance homeostasis model assessment; AUC_INS_, area under curve of insulin concentration*.

**Table 4:** **Effect of the ET_A_ receptor genotypes on clinical characteristics of Japanese discordant siblings before and after adjustment for age, gender, and BMI (Mean ± SD)**.

Traits	Genotypes		Unadjusted *p*-value	Adjusted *p*-value
	TT/TC	CC	
Subject number	316	29		
Blood pressure (mmHg)
Systolic	131.9 ± 1.2	132.8 ± 3.7	0.9057	0.8309
Diastolic	78.1 ± 0.7	79.3 ± 2.1	0.8858	0.6028
Lipid profiles (mg/dl)
Triglycerides	168.9 ± 6.7	138.0 ± 20.1	0.1486	0.1353
Total CHOL	198.9 ± 2.4	199.4 ± 6.9	0.8455	0.9426
HDL-cholesterol	48.4 ± 0.9	54.9 ± 2.6	0.0461	0.0155
VLDL-cholesterol	31.6 ± 1.1	24.8 ± 2.9	0.0450	0.0255
LDL-cholesterol	118.1 ± 2.1	114.4 ± 6.5	0.4378	0.5769
Atherogenic index (CHOL/HDL)	4.4 ± 0.1	3.8 ± 0.2	0.0273	0.0214
Oral glucose tolerance test
Plasma glucose (mg/dl)
Fasting	96.8 ± 0.6	98.8 ± 1.8	0.2310	0.2882
1-h	166.6 ± 3.5	167.0 ± 9.7	0.9227	0.9662
2-h	139.7 ± 3.0	146.2 ± 8.8	0.5161	0.4716
Plasma insulin (μIU/ml)
Fasting	7.3 ± 0.3	6.1 ± 0.8	0.6009	0.1256
1-h	63.1 ± 3.0	65.4 ± 9.1	0.4749	0.8081
2-h	63.4 ± 3.1	59.7 ± 9.5	0.9894	0.6996
Insulin resistance indices
HOMA_IR_	1.8 ± 0.1	1.5 ± 0.2	0.8718	0.2348
AUC_INS_ (μIU/ml/h)	97.9 ± 4.2	99.8 ± 12.7	0.5342	0.8859

*CHOL, cholesterol; HDL, high density lipoprotein; LDL, low density lipoprotein; VLDL, very low density lipoprotein; HOMA_IR_, insulin-resistance homeostasis model assessment; AUC_INS_, area under curve of insulin concentration*.

Since phenotypic expression of IR-associated hypertension is diverse and bounded, we further performed correlation analysis by treating the indices of insulin sensitivity as dependent variables, and the potential contributors including the ET_A_ receptor genotype, BMI, age, et cetera as independent variables. By this approach we could predict if any attribute, while controlling for other covariates, is independently associated with changes in the indices at all. Results on the levels of significance in the pooled studied subjects are present in Table 5. The correlations among interrelating IR traits for example BMI and waist circumference showed significance as would be expected. In particular, we found that ET_A_ receptor T323C genotype was not only significantly (*p* = 0.0012) but also independently from BMI, waist circumference, triglycerides and HDL-cholesterol concentrations, and gender correlated with the index of AUC_INS_. Additionally, we observed that waist circumference turned into a more significant predictor than WHR to HOMA_IR_ and AUC_INS_ in our studied population.

**Table 5:** **Results on the *p*-values of correlation analysis using insulin sensitivity indices as dependent variables and insulin resistance related traits and genotypes as independent variables**.

Independent traits	Dependent variable	
	HOMA_IR_	AUC_INS_
ET_A_ receptor genotype (TT + TC vs. CC)	NS	0.0012
Covariate
Blood pressure	NS	NS
Body mass index	<0.0001	<0.0001
Waist-to-hip ratio	NS	NS
Waist	0.0002	0.0008
Triglycerides	<0.0001	<0.0001
HDL-cholesterol	<0.0001	<0.0001
LDL-cholesterol	NS	NS
VLDL-cholesterol	NS	NS
Age	NS	NS
Gender	0.0001	0.0001

*HOMA_IR_, insulin-resistance homeostasis model assessment; AUC_INS_, area under curve of insulin concentration*.

## Discussion

Enhanced vascular gene expressions of ET-1 and ET_A_ receptor, and an amelioration of hypertension via administration of selective ET_A_ receptor antagonist observed previously in our fructose-induced hypertensive rats (18) and in comprehensive reviews (15, 37, 38) suggested that ET_A_ receptor may play a role in the regulation of BP and consequently its antagonists may become one of the therapeutic strategies in clinical. To date, genetic variations and association studies of this putative hypertension gene (23–25, 29, 39) have been performed mostly in subjects of European ancestry but not in Asian origins excepta few literature regarding Japanese (26, 40). Therefore, we conducted this study to investigate whether the T323C polymorphism of the gene encoded for ET_A_ receptor might be involved in the predisposition in IR-associated hypertension in a large Asian cohort ascertained via essential hypertension. The novel findings of this study are firstly the observation of noticeable lower minor allele frequency in our overall Asian subjects compared to multi-national population reported in gene database (http://www.genecards.org) at 0.22 and 0.35, respectively. Secondly, an ethnic difference between Chinese and Japanese we studied on the allele frequencies of the T323C variant was identified. Thirdly, the ET_A_ receptor T323C polymorphism was associated with discrete features of the MetS in our two Asian populations.

Although significant differences on the genotypic and allelic frequencies of this T323C variant of the ET_A_ receptor gene were observed between our subjects of Chinese and Japanese origin, no association of the T323C polymorphism with hypertension status was seen in either Asian ethnic group, i.e., neither systolic nor diastolic BP appeared to be influenced by the gene variant. Our findings supported and extended the prior observations in Caucasians (23, 24, 29) that there is no significant association of this solely gene polymorphism with BP. On the contrary, some other polymorphisms of ETS have been shown to be associated with BP either directly or in interaction with other genes/factors, for example C1363T in exon 8 of the ET_A_ receptor on pulse pressure of normotension (24), G198T in exon 5 of the ET-1 gene together with BMI on BP (26), and A985G in 3′-untranslated region of the ET-2 gene on the severity of hypertension (41). Moreover, one association study reported a significant interaction of ET_A_ receptor T323C and ET-1 138 insertion/deletion polymorphisms on diastolic BP in normotensive subjects (25).

Thus far, the predisposition of genes involved in ETS has been illustrated in the CVD including hypertension (23, 25, 26, 41), myocardial infarction (24), cardiomyopathy (39), and others (42). To our knowledge, no study has been conducted to determine the implication of the T323C polymorphism of ET_A_ receptor gene in IR, an intermediate metabolic phenotype for hypertension (43). Thus, we further explored the association of the ET_A_ receptor’s T323C polymorphism with the MetS related quantitative traits. A noteworthy finding in our study was that Chinese siblings who were carriers of the C homozygote, the rare allele of ET_A_ receptor T323C polymorphism, manifested characteristics of IR, namely significantly higher levels of fasting insulin, HOMA_IR_ score, and AUC_INS_ at 29.3, 35.3, and 39.3%, respectively, when compared to their counterparts with TT/TC genotypes. Consistent with the observation, results from regression analysis revealed that the ET_A_ receptor T323C genotype was a significant determinant for AUC_INS_ independent from confounders in our pooled Asian population. Meanwhile, we observed that the CC genotype was significantly associated with an increased level of HDL cholesterol at 13.4% and consequently with a reduced ratio of Total/HDL cholesterol in Japanese. The current findings suggested a recessive effect of allele C in the ET_A_ receptor T323C polymorphism on an adverse insulin response in Chinese and a favorite atherogenic index in Japanese.

Numerous studies provide convincing evidence that phenotypic aspects of MetS differ among ethnicities. Since MetS involves a constellation of highly intercorrelated quantitative phenotypes, factor analysis has been applied in attempts to simplify the risk factors and to discover the underlying pathophysiological mechanisms (44). Evaluation of the MetS in multi-ethnic groups including the SAPPHIRe participants has shown that the identified factor pattern was very similar in African Americans, Hispanics, Whites, and Japanese, but not entirely so in Chinese (45). The common factor, interpreted as obesity, explained the largest and comparable portion of the overall variance in both the Chinese and the Japanese though the factor loadings themselves were substantially different in magnitude between the two ethnic groups. On the other hand, insulin level was the major component of the second factor and in together with other variables contributed nearly 17% of the variance in Chinese, but insulin was merely an intermediate component for Japanese. Conversely, the lipid factor, characterized by a negative loading for HDL cholesterol, was more prominent in Japanese than in Chinese. These results suggest ethnic variability in susceptibility to the specific risk factors of the MetS, which strengthens our findings that the T323C polymorphism was associated with not only the distinct but the more prominent phenotype for each ethnic group. However, the mechanisms that this ET_A_ receptor T323C polymorphism might exert its opposite influence on MetS in different racial people are not yet elucidated in this study.

Human genome contains a single copy of the ET_A_ receptor gene which spans over 64 kb on chromosome 4q31.22 and comprises eight exons and seven intros. Though examining sole single nucleotide polymorphism might have limitations of interpretation, study on combination of four common haplotype blocks merely revealed mild effect on the risk of hypertension (29). It should be noted that this variant located within codon 323 in the sixth exon of ET_A_ receptor gene is a silent polymorphism (CAT → CAC, His → His). As a silent polymorphism which nucleotide substitution on the third base of the codon does not alter the amino acid sequence of the receptor protein, it is unlikely that the variant itself is involved in the pathophysiology of MetS. One possibility for this silent polymorphism exerts its influence is that it is in linkage disequilibrium with a nearby causal variant which has not yet been identified. In addition, the wobble base pair is a fundamental unit for RNA secondary and tertiary structures and therefore may have functional consequences. Without further RNA studies, the possibility of unrecognized alleles, disrupting exonic splicing enhancers, and/or influencing gene transcription cannot be excluded. Although BP and IR have been shown to cosegregate in Hispanic families with a hypertensive proband (6), no correlation was observed between BP and insulin sensitivity index in our Asian subjects. The lack of relationship may partially be due to the fact that the measurement of BP of the hypertensive individuals in SAPPHIRe study was confounded by medication since majority of our hypertensive subjects were on antihypertensive treatment. In addition, our BP measurements feasible for large-scale study may not be sensitive enough as 24 h ambulatory recordings, a necessary tool to identify a relationship between BP and IR within the hypertensive range suggested by Nilsson et al. (46).

Beyond candidate genes in ETS for the predisposition of IR-associated hypertension, the notion of using biological candidate genes for interrelating traits as genetic markers for the features of MetS and/or diabetes has also been applied in other studies regardless successes or not. For example, Bayoglu et al. identified polymorphism rs10757278 on chromosome 9p21 which is risk locus for coronary artery disease may confer increased risk for MetS in Turkish people (47). Ned et al. merely found marginal association between inflammation-related genes and the susceptibility of albuminuria (48), and Moon et al. on the other hand, were unable to relate polymorphisms of pyruvate dehydrogenase kinase four gene, a mitochondrial kinase attributing to hyperglycemia, with MetS or diabetes in Korean (49). One example of linking candidate gene deletion in mice with a polymorphism association study in patients with hypertension has shown that rs10491334 of calcium/calmodulin-dependent kinase IV gene may play a pivotal role in hypertension via the regulation of endothelial nitric oxide synthase (50). Another example of candidate genotype polymorphism in a small sample sized population with a replication of another same ethnic population revealed a significant association of the platelet antigen variant of glycoprotein IIIa (PI^A1/A2^) with stroke in a sub-group of high risk hypertension patients (51). Moreover, recent genome-wide association studies (GWAS) have identified susceptibility loci for various component traits of MetS including lipid/lipoprotein profiles (52, 53) and obesity (54), or for MetS as an entity in European ancestry (52, 55) and Indian Asian (56). Given these research findings affirm the multifactorial aetiology and multigenic nature of MetS. However, these GWAS approach in combination with either bi- or multi-variate haplotypes done in Asian ethnic’s remains to be seen (55). A trans-ethnic GWAS with fine mapping of these genotypes with optimal sample size may find population specific associations (57) and a similar replication study conducted in a separate Chinese–Japanese population may be advised in further works (58).

In conclusion, we have successfully related the T323C polymorphism of ET_A_ receptor gene to features of MetS in this study though no association between the gene variant and BP is reassuring in our hypertensive subjects of Chinese and Japanese origin. Our findings can be considered as the first time to be related directly to IR, and also the first in Asian populations despite its dissociated effects on different ethnicities. If reproducible in other independent ethnicities, our results reported herein suggest that the polymorphism of ET_A_ receptor gene may serve as one of the independent determinants for manifested IR which is often a prelude to hypertension. Nevertheless, the functional significance of this particular gene variant, the inference on haplotype effects, and the gene–gene/gene-environment interactions remain to be elucidated. Determinants of CVD risk in diabetes beyond hyperglycemia has been an important issue to address lately, since intensive control of plasma sugar alone is often insufficient to reduce the incidence of major CVD in these patients (59). Components of MetS and many other CVD risk factors including those with pro-inflammatory, pro-thrombotic, and/or oxidative stress effects all need to be considered and tackled in addition to control of hyperglycemia. Among them, ET-1, the key member of ETS, by playing multiple roles relating IR, hypertension, as well as proliferative and pro-inflammatory effects may be a perspective target for drugs discovery and/or other intervention modalities useful not only for management but prevention of MetS, diabetes mellitus and related CVD (38).

## Authors Contribution

Guarantor of integrity of the study and study concepts: Low-Tone Ho. Definition of intellectual content: Low-Tone Ho, John Grove, Thomas Quertermous. Literature research: Yung-Pei Hsu, Chi-Chung Juan, Low-Tone Ho. Clinical studies: Low-Tone Ho, Chih-Tai Ting, Kuang-Chung Shih, Lee-Ming Chuang, Kamal Masaki. Genotyping experiments: Yung-Pei Hsu, Chi-Chung Juan. Data acquisition: Chin-Fu Hsiao, Ming-Wei Lin, Yung-Pei Hsu, Chi-Chung Juan. Statistical analysis: Chin-Fu Hsiao, Ming-Wei Lin, Shu-Chiung Chiang. Manuscript preparation: Yung-Pei Hsu, Low-Tone Ho. Manuscript editing and revise: Low-Tone Ho, Yii-Der I. Chen.

## Conflict of Interest Statement

The authors declare that the research was conducted in the absence of any commercial or financial relationships that could be construed as a potential conflict of interest.

## Abbreviations

AUC_INS_, area under curve of insulin concentration; BMI, body mass index; BP, blood pressure; CAP, College of American pathologists; CDC/NHLBI, centers for disease control and prevention/national heart, lung, and blood institute; CVD, cardiovascular disease; ET-1, endothelin-1; ET_A_ receptor, endothelin type A receptor; ET_B_ receptor, endothelin type B receptor; ETS, endothelin system; GWAS, genome-wide association studies; HDL, high density lipoprotein; HOMA_IR_, insulin-resistance homeostasis model assessment; IR, insulin resistance; LDL, low density lipoprotein; MetS, metabolic syndrome; OGTT, oral glucose tolerance test; PCR, polymerase chain reaction; RFLP, restriction fragment length polymorphism; SAPPHIRe, Stanford Asian-Pacific program in hypertension and insulin resistance; VLDL, very low density lipoprotein; WHR, waist/hip ratio.
